# BELIEFS SURROUNDING THE PAIN AND DEPRESSION CYCLE AMONG OLDER AFRICAN AMERICAN WOMEN: FINDINGS FROM FOCUS GROUPS

**DOI:** 10.1093/geroni/igz038.2256

**Published:** 2019-11-08

**Authors:** Emerald Rivers, Brittany Drazich, Sarah Szanton, Manka Nkimbeng, Janiece Taylor

**Affiliations:** Johns Hopkins University, Baltimore, Maryland, United States

## Abstract

Older African American women experience high rates of comorbid conditions and functional limitations that put them at risk of experiencing a cycle of pain and depressive symptoms. This cycle is often shaped by individual’s behaviors, emotions, physical responses, and thoughts. Increased pain severity is associated with comorbid pain and depression making it essential for older African American women to communicate their experiences with these conditions. Hence, we explored older adult African American women’s relevant beliefs, and identified strategies to address them in adapting the intervention, Get Busy Get Better. In three focus groups, we found that older African American women (mean age 60.7, n=11): (1) relied on companionship (emotions), (2) used physical activity strategies for pain and depression relief (behaviors), (3) had a general function reduction from pain (physical response), and (4) saw connections between depression and pain (thoughts). Thus, when adapting the intervention, strategies incorporate these four elements.

